# The mediating role of health literacy in the relationship between self-care and planned behavior against Covid-19

**DOI:** 10.1186/s12879-024-09513-8

**Published:** 2024-06-20

**Authors:** Sirous Panahi, Hossein Ghalavand

**Affiliations:** 1 Department of Medical Library and Information Science, School of Health Management and Information Sciences, Iran University of Medical Sciences, Tehran, Iran; 2 Department of Medical Library and Information Science, Abadan University of Medical Sciences, Abadan, Iran

**Keywords:** Health literacy, Self-care, Theory of planned behavior, Covid-19, Pandemic

## Abstract

**Background:**

Planned behaviors and self-care against the coronavirus are two important factor in controlling its spread and self-care behaviors depend on the level of health literacy. This research was conducted to determine the mediating role of health literacy in the relationship between elements of planned behavior and self-care in dealing with the Covid-19.

**Methods:**

In this descriptive-analytical quantitative study, the sample size was calculated using Cochrane’s formula and considering a p-value of 0.51, α = 0.05, and d = 0.05, and 313 students were selected based on stratified and random method. To gather data and assess various aspects of variables, a questionnaires were utilized, focusing on health literacy, self-car and planned behavior. The relationship between the variables was examined by SPSS version 26 and via descriptive statistics, including the mean and standard deviation, and inferential statistics such as Pearson’s correlation coefficient (*P* = 0.05), path analysis, and determining the standard coefficients between self-care and planned behavior, mediated by the indicators of the health literacy.

**Results:**

A significant difference was found between the level of health literacy of women and men. The comparison of the mean health literacy and self-care behavior in terms of other variables did not show any significant difference. Meanwhile, the comparison of health status control behaviors, hand washing, and mask use did not show any significant difference between the two groups. A positive and significant correlation was found between self-care behaviors, attitude, subjective norms, perceived behavioral control, and behavioral intention. The relationship of health literacy and psychological variables of attitude, subjective norms, and perceived behavioral control with self-care against COVID-19 was significant.

**Conclusion:**

The direct and significant impact of health literacy on individuals’ self-care behaviors against the coronavirus was not observed. However, health literacy did have a significant effect on subjective norms. This finding is important because subjective norms significantly influenced individuals’ behavioral intention, which in turn had a significant effect on self-care behaviors against the coronavirus. Thus, health literacy played a mediating role in this relationship. Furthermore, attitude emerged as the strongest predictor of behavioral intention, exerting a direct effect. Conversely, perceived behavioral control did not directly and significantly affect students’ self-care behaviors.

**Supplementary information:**

The online version contains supplementary material available at 10.1186/s12879-024-09513-8.

## Background

Self-care is a key control approach and a cognitive activity whereby people play a major role in maintaining their health. People’s ability to take care of themselves and adhere to the recommended protocols is the main method of preventing infection with the coronavirus [1, 2]. Self-care against the coronavirus includes actions such as observing social distancing and wearing a mask. Awareness and adherence have played a significant role in controlling the COVID-19 pandemic. Self-care involves acquired, conscious, and purposeful actions that people undertake for the health of themselves, their children, and their families [3, 4].

There is a direct link between self-care, adherence to medical and health recommendations, and health literacy [5]. Health literacy refers to people’s ability to receive, process, and comprehend health information, which can lead to better decision-making at different times [6, 7]. It is a factor influencing people’s self-care for disease control and prevention. People’s low health literacy level and their inability to understand the information provided by health professionals can negatively impact their health and increase their medical expenses [4]. Accordingly, measuring health literacy can contribute to detecting people’s abilities and designing necessary educational interventions to improve their health literacy [8]. Therefore, paying attention to the link between health literacy and self-care against the coronavirus can prove a proper strategy to support preventive activities during the outbreak of such infectious diseases.

Given people’s health-related and behavioral problems in dealing with health challenges, theories and behavioral patterns can be employed to determine and identify the factors affecting health-related behaviors [9]. In fact, the use of theories to describe people’s behavior during health crises can enhance the efficiency, effectiveness, and chance of success in obtaining the desired outcomes [10]. Various guidelines and recommendations were emphasized during the COVID-19 outbreak, and people were expected to play a key role in self-care and control the spread of the virus by adhering to these guidelines; in practice, however, some communities did not succeed in this regard. There are various theories about health-related behaviors whose core deals with doing or not doing predetermined behaviors [11–13]. In the sphere of health, the theory of planned behavior (TPB) is a theory of behavior change whose efficiency and effectiveness have been proven in previous studies [14, 15]. TPB asserts that individual beliefs regarding a specific behavior influence their attitude towards it, the prevailing subjective norms, and the perceived behavioral control, ultimately leading to the intention to engage in that behavior. TPB incorporated the concept of behavioral control as a crucial determinant of health behavior, alongside attitude and subjective norms. TPB establishes a causal chain linking beliefs (behavioral, normative, and control beliefs) to intentions and behaviors through attitudes, norms, and perceived control, providing a structured approach to identify key factors influencing an individual’s decision-making process. Given the changeability of beliefs and attitudes, they serve as prime targets for interventions aimed at modifying behavior [14–17].

In TPB, the main construct that determines behavior is the person’s intention, and the three constructs of attitude, subjective norms, and perceived behavioral control affect this intention [18]. Based on the TPB, the more favorable one’s attitude towards a behavior, the more likely the intention to perform the behavior. In this theory, subjective norms include a person’s subjective perception of others’ approval or disapproval of performing a behavior, and membership in support groups and increasing social support can lead to performing or not performing a certain behavior. Perceived behavioral control is the degree of control one feels to perform or not perform a behavior, which has a significant association with personal will [19, 20]. The TPB has been employed in the domain of health, especially in patients’ self-care, and its efficacy has been confirmed in predicting and comprehending healthy and unhealthy behaviors and their related outcomes [1, 2, 5, 15, 19].

Overall, paying attention to planned behaviors and self-care against the coronavirus is a major factor in controlling its spread, and self-care behaviors depend on the level of health literacy. Although some studies have been conducted on health literacy and self-care behavior for different diseases [4, 11, 13, 21]. This study was planned to determine the relationship between health literacy and self-care behavior, mediated by the constructs of planned behavior, was examined among students of Abadan University of Medical Sciences (Iran).

## Methods

### Population and samples

This descriptive-analytical quantitative study was conducted to determine the relationship between health information literacy, elements of planned behavior, and self-care in dealing with the coronavirus. The research population included all students studying at Abadan University of Medical Sciences (AbadanUMS) in the academic year 2022–2023 (*n* = 1698). The sample size was calculated using Cochrane’s formula and considering a p-value of 0.51, α = 0.05, and d = 0.05, and 313 students were selected, divided by their fields of study. Sampling was stratified and random.

### Measurement

A four-part questionnaire was used to collect data. The first part include demographic characteristics such as sex, age, and a history of contracting the coronavirus. In the second part, to measure health literacy, the short form of the standard Health Literacy Questionnaire was used as the most common and comprehensive standard instrument for measuring health literacy [22]. This questionnaire has 33 items based on a Likert scale (from 1 = never to 5 = always); its validity is 0.83 (Cronbach’s coefficient), and its reliability has been confirmed (coefficient of 0.93) in previous studies [19, 20]. In the third part, to measure the constituents of the TPB, 17 questions in four groups were considered to measure the scales of attitudes (Q1-Q3), subjective norms (Q4-Q6), perceived behavioral control (Q7-Q9), and behavioral intention (Q10-Q17), based on a five-point Likert scale (1 = completely agree to 5 = completely disagree). The content validity index (CVI = 0.83) and content validity ratio (CVR = 0.86) confirmed the face and content validity of this part of the questionnaire [23, 24]. In the fourth part, a literature review was conducted, the accepted international protocols for self-care and preventing the spread of the coronavirus were extracted, and experts were consulted. This questionnaire include social distancing (Q1, Q7), vaccine injection (Q8), check health status (Q5, Q6), washing hands (Q3, Q4) and Use a disposable mask (Q2). The electronic form of this questionnaire was designed, and a link to it was sent to the participants.

### Statistical analysis

SPSS (v. 26) was used for data analysis. The association between the variables was examined via descriptive statistics, including the mean and standard deviation (SD), and inferential statistics such as Pearson’s correlation coefficient (*P* = 0.05), path analysis, and determining the standard coefficients between self-care behaviors and health literacy, mediated by the indicators of the TPB.

## Results

A total of nine student from the selected samples did not participate in this study. Table 1 shows the level of health literacy and self-care behavior based on different demographic characteristics of the 305 participants. Most of the participants were women, aged 18–20 years, and were seniors. A significant difference was found between the level of health literacy of women and men, where women had a higher mean health literacy. Besides, there was a significant difference in the mean health literacy of the students based on the academic semester, and the level of health literacy increased with the semesters. The comparison of the mean health literacy and self-care behavior in terms of other variables did not show any significant difference.

**Table 1:** Comparing the level of health literacy and self-care behaviors based on demographic characteristics

Variable		Frequency (Percent)	Health literacy (Mean ± SD)	Significant level	Self-care behavior (Mean ± SD)	Significant level
Gender	Male	149 (48.9)	45.74 ± 12.7	*P* = 0.030	3.99 ± 0.73	*P* = 0.457
	Female	156 (51.1)	52.33 ± 13.51		4.04 ± 0.81	
Age	18–20 year	142 (46.6)	46.66 ± 14.93	*P* = 0.413	4.43 ± 1.48	*P* = 0.107
	21–23 year	119 (39.00)	46.47 ± 17.15		4.36 ± 1.09	
	24–26 year	33 (10.8)	47.79 ± 14.67		4.37 ± 1.38	
	More than 27 year	11 (3.6)	52.80 ± 13.54		4.09 ± 1.37	
Semester	1–2	30 (9.84)	39.91 ± 10.16	*P* = 0.015	3.78 ± 0.53	*P* = 0.425
	3–4	63 (20.62)	44.39 ± 11.13		4.07 ± 0.89	
	5–6	71 (23.48)	45.16 ± 13.57		4.14 ± 0.43	
	More than 7	141 (46.08)	54.33 ± 14.10		4.11 ± 0.88	
Marital Status	Single	250 (82.14)	47.01 ± 14.03	*P* = 0.724	3.89 ± 0.82	*P* = 0.868
	Married	54 (17.86)	47.66 ± 11.66		3.98 ± 0.69	
Economic status	Good	72 (23.70)	46.41 ± 13.7	*P* = 0.301	4.08 ± 0.61	*P* = 0.161
	Medium	167 (55.01)	46.21 ± 14.2		4.02 ± 0.63	
	Weak	64 (21.29)	50.02 ± 11.6		3.85 ± 0.82	
frequency of covid-19 infection	0	93 (30.51)	46.43 ± 14.93	*P* = 0.725	4.43 ± 1.58	*P* = 0.317
	1–2	142 (46.7)	47.97 ± 12.15		4.36 ± 1.39	
	3–4	45 (14.69)	47.40 ± 11.47		4.07 ± 1.49	
	More than 4	24 (8.1)	49.35 ± 12.80		4.09 ± 1.37	

In this research, the mean comparison test was used for two independent groups of men and women. Based on Table 2, the mean of attitude, subjective norms, and behavioral intentions differed between men and women based on the levels of health literacy. The subscales related to students’ self-care showed a significant difference between men and women based on their compliance with social distancing and vaccination. Meanwhile, the comparison of health status control behaviors, hand washing, and mask use did not show any significant difference between the two groups.

**Table 2:** Comparison of mean and standard deviation of TPB constructs and self-care behaviors according to health literacy

Variable		Low health literacy (Mean ± SD)	Adequate health literacy (Mean ± SD)	Significant level
TBT subscales	Attitudes	4.56 ± 0.50	4.90 ± 0.57	*P* = 0.000
	Subjective Norms	4.78 ± 0.68	4.95 ± 0.56	*P* = 0.000
	Perceived Behavior control	4.07 ± 0.66	4.42 ± 0.75	*P* = 0.570
	Preventive behavior	4.59 ± 0.79	4.92 ± 0.68	*P* = 0.000
Self-care subscales	Social distancing	4.42 ± 0.90	4.75 ± 0.87	*P* = 0.008
	Vaccine injection	2.90 ± 1.91	3.82 ± 1.36	*P* = 0.018
	Check health status	4.36 ± 0.80	4.65 ± 0.85	*P* = 0.221
	Washing hands	3.07 ± 1.44	2.71 ± 1.43	*P* = 0.180
	Use a disposable mask	2.75 ± 0.94	2.66 ± 0.82	*P* = 0.092

Based on Table 3, a positive and significant correlation was found between self-care behaviors, attitude, subjective norms, perceived behavioral control, and behavioral intention.

**Table 3:** Pearson correlation coefficient between health literacy, TBP constructs and self-care behaviors

Variable	1	2	3	4	5	6	7	8
1- Age	1							
2- Frequency of infection	0.59*	1						
3- Health Literacy	0.22*	0.21*	1					
4- Attitudes	0.21	0.11	0.12	1				
5- Subjective Norms	-0.09	0.07	0.14*	0.34**	1			
6- Perceived Behavior Control	0.11	0.10	0.28**	0.29*	0.16	1		
7- Preventive behavior	0.14	0.30*	0.17*	0.42**	0.19**	0.24**	1	
8- Self-care Behavior	0.17	0.15	0.12	0.37**	0.25**	0.20**	0.33**	1

*P* < 0.01*, *P* < 0.05**

**Fig. 1:**
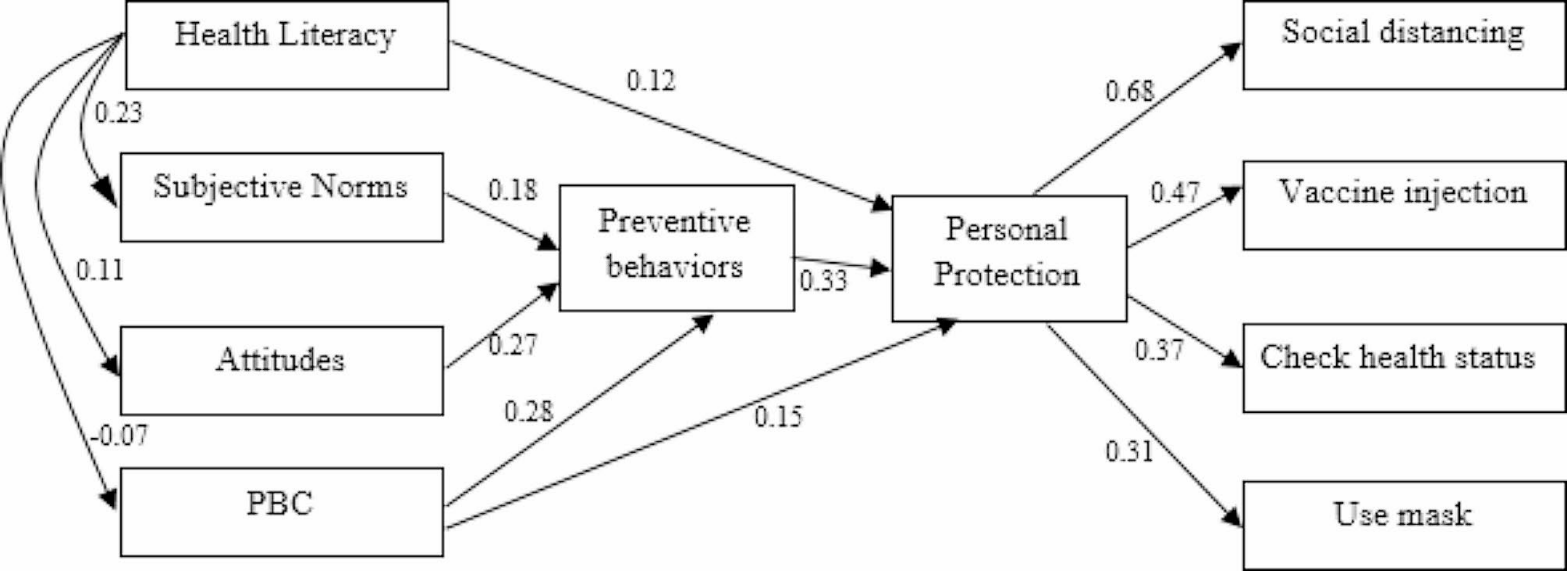
Path analysis and standardized coefficients between constructs of TBP, self-care behaviors and health literacy

Figure 1 displays the results of path analysis and standard coefficients. Health literacy did not have a direct and significant effect on self-care behaviors against the coronavirus. Still, its effect on subjective norms was significant, and due to the significant effect of subjective norms on behavioral intention and the significant effect of behavioral intention on self-care against the coronavirus, health literacy was a mediator variable. Moreover, attitude was the greatest predictor of behavioral intention directly; perceived behavioral control did not directly and significantly affect the students’ self-care, but its effect was mediated by behavioral intention. The fit indices of the model (Fig. 1) indicate the fit of the data to the model. In general, the model predicted 0.346 of the variance of the final variable, i.e., self-care against COVID-19.

## Discussion

The findings revealed that there is a significant difference between the mean health literacy of male and female students. Women are more literate in understanding medical forms, medication usage instructions, and written information, and the level of health literacy between women and men may be different in various social strata and cultures [25–27]. Men make less effort to obtain information due to subjective beliefs, lower perceived sensitivity to illness, and less understanding of health threats. This difference can possibly make women more willing to report diseases compared to men [5, 27, 28]. Not having enough time to search for health information, especially when the disease is not quite threatening or serious, could be another reason for the low level of health literacy in men [29].

The findings of the present study demonstrated a significant difference in health literacy between the participants based on academic semesters. In general, the power of recognition and understanding to comprehend health literacy increases with the level of education. People’s problems with using different media, along with their little familiarity with medical terms, can have a negative impact on their ability to interact successfully with healthcare systems [25, 30]. The ability to access simplified health information is another factor in improving health literacy. The use of simple images and proper examples can facilitate people’s understanding of health-related topics [31, 32]. It is necessary for healthcare systems to modify their information services according to people’s health literacy level and provide training through simple strategies such as face-to-face counseling, group discussions, and educational pamphlets [23, 33].

The results of this research showed a significant difference in the mean attitude score between the two groups with a low and adequate health literacy level. Positive attitudes towards self-care and adherence to correct health-related behaviors are crucial, and there is a direct relationship between health literacy and attitudes [34]. Positive attitudes and a high level of health literacy encourage patients to make appropriate decisions. When people feel that a behavior leads to a positive outcome, they adopt and maintain that behavior [35].

The difference in subjective norms scores between students with the health literacy level was another finding of this study. In diseases such as diabetes, the patient’s family can play a central role in the administration of self-care training methods. Patients whose families have adequate information about the disease and recommend correct health-related behaviors have more effective control and better compliance with treatment [35, 36]. As a result, the formation of support groups and the participation of important people, such as the family, in self-care programs can help promote the health level of patients by strengthening the mentality of support and confirming the continuation of correct health-related behavior [37–39].

The findings of this research revealed a significant relationship between perceived behavioral control, health literacy level, and self-care against the coronavirus. Perceived behavioral control refers to a person’s judgment about being under control and their intentional ability to perform a specific action, which is an important factor in their performance. Perceived behavioral control is a key predictive factor in people’s intention to perform health-related behaviors and can be increased by creating a suitable environment to acquire the skills and knowledge required for behavioral control and personal empowerment [21, 36]. People with a low level of behavioral control make less effort to perform the right health-related behaviors or change wrong behaviors [7, 31, 38]. Modeling, repeating in practice, simplifying, and dividing a behavior into smaller steps, as well as strategies such as goal-setting, planning action, and planning to overcome obstacles, will ultimately have a positive effect on self-care [40–42].

Results of the present study, like previous studies, demonstrated a significant difference between the two groups of participants with poor and adequate health literacy in terms of self-care behaviors, including social distancing and vaccination [26, 43, 44]. Those with low health literacy are less likely to understand written and spoken information provided by healthcare professionals and follow their instructions. These people have a worse health status, a higher rate of hospitalization, more visits to the doctor, and weaker self-care skills [30, 40, 41]. In general, people with a low level of health literacy often use passive communication methods, do not participate in decision-making, and face numerous problems in interacting with their physicians [5, 13, 45]. Therefore, healthcare professionals should empower people and patients through various trainings to improve their self-confidence, increase their participation, and help them establish effective communication with healthcare providers. In fact, self-care is based on knowledge and is influenced by people’s health-related knowledge. The higher people’s health-related knowledge, the better their ability to identify self-care needs, plan how to meet these needs, and make judgments and decisions about prioritizing their needs [35, 46].

In the present study, the TBP theory constructs predicted 0.346 of self-care behaviors. Regression analysis in previous studies showed that 41.5% of the variance of intention and 26.2% of the variance of behavior was predicted by the constructs of TBP theory [47]. Furthermore, similar to the findings of other studies, attitude was the most important predictor of self-care behavior in students during the COVID-19 outbreak [8, 26, 35, 46]. The severity and sensitivity of complications, costs, and benefits of following self-care can be a major part of the behavior variance [48, 49].

## Conclusion

This study investigated the mediating role of health literacy in the relationship between the TPB, and self-care behaviors against the coronavirus among the students of Abadan University of Medical Sciences. The results revealed that the health literacy of female students was higher than that of male students. The relationship of health literacy and psychological variables of attitude, subjective norms, and perceived behavioral control with self-care against COVID-19 was significant.

The present study was not possible to obtain and analyze causal relationships due to budget and time constraints. The students filled out the instruments as self-reports, and there is a possibility of bias in completing the questionnaires. The researcher’s lack of complete control over the participants and their follow-up of, especially regarding the observance of health recommendations related to the coronavirus, was the other limitation of this research. The students of a single university participated in this study, and the generalization of the results is very difficult and limited. As such, it is recommended that similar studies be conducted in other regions and for other diseases. Although the effectiveness of the TBP theory was proven in predicting and determining the factors affecting self-care behaviors during the COVID-19 outbreak, it should be noted that behavior is a multidimensional and multifactorial issue. Therefore, it is suggested that other psychological variables in the form of behavior change models and theories be used in future studies to explore and predict the relationship between self-care and health literacy.

### Electronic supplementary material

Below is the link to the electronic supplementary material.

Supplementary Material 1

## Data Availability

The datasets used and analysed during the current study are available from the corresponding author on reasonable request.

## Abbreviations

TBT: Theory of Planned Behavior

## References

[1] Cindioglu C, Beyazgul B, Koruk I. Is coronavirus–COVID-19 stress effective in self-protection behavior? International Medicine, 2021: pp. 10–15.

[2] Zheng D, Luo Q, Ritchie BW (2021). Afraid to travel after COVID-19? Self-protection, coping and resilience against pandemic ‘travel fear’. Tour Manag.

[3] Davies N (2011). Promoting healthy ageing: the importance of lifestyle. Nurs Standard (through 2013).

[4] Sørensen K (2012). Health literacy and public health: a systematic review and integration of definitions and models. BMC Public Health.

[5] Khakzadi H, Morshedi H. Association between Health Literacy and theory of Planned Behavior with Self-Care behaviors in type 2 Diabetic patients. Volume 6. Journal of Torbat Heydariyeh University of Medical Sciences; 2019. pp. 33–46. 4.

[6] Ratzan S (2000). National library of medicine current bibliographies in medicine: health literacy.

[7] Rezakhani Moghaddam H, Ranjbaran S, Babazadeh T (2022). The role of e-health literacy and some cognitive factors in adopting protective behaviors of COVID-19 in Khalkhal residents. Front Public Health.

[8] Powers BJ, Trinh JV, Bosworth HB (2010). Can this patient read and understand written health information?. JAMA.

[9] Wollast R (2021). The theory of planned behavior during the COVID-19 pandemic: a comparison of health behaviors between Belgian and French residents. PLoS ONE.

[10] Fan C-W, et al. Extended theory of planned behavior in explaining the intention to COVID-19 vaccination uptake among mainland Chinese university students: an online survey study. Volume 17. Human Vaccines & Immunotherapeutics; 2021. pp. 3413–20. 10. 10.1080/21645515.2021.1933687 PMC8437493 34170792

[11] Okan O, et al. Health literacy as a social vaccine in the COVID-19 pandemic. Health Promotion International; 2022. 10.1093/heapro/daab197 PMC8807235 35022721

[12] Turhan Z, Dilcen HY, Dolu İ (2022). The mediating role of health literacy on the relationship between health care system distrust and vaccine hesitancy during COVID-19 pandemic. Curr Psychol.

[13] Yusefi AR (2022). Health literacy and health promoting behaviors among inpatient women during COVID-19 pandemic. BMC Womens Health.

[14] Lucarelli C, Mazzoli C, Severini S (2020). Applying the theory of planned behavior to examine pro-environmental behavior: the moderating effect of COVID-19 beliefs. Sustainability.

[15] Shmueli L (2021). Predicting intention to receive COVID-19 vaccine among the general population using the health belief model and the theory of planned behavior model. BMC Public Health.

[16] Paul B et al. A systematic review of the theory of planned behaviour interventions for chronic diseases in low health-literacy settings. J Global Health, 2023. 13. 10.7189/jogh.13.04079 PMC10506128 37681679

[17] Sheeran P (2016). The impact of changing attitudes, norms, and self-efficacy on health-related intentions and behavior: a meta-analysis. Health Psychol.

[18] Gibson LP (2021). Theory of planned behavior analysis of social distancing during the COVID-19 pandemic: focusing on the intention–behavior gap. Ann Behav Med.

[19] Yahaghi R (2021). Fear of COVID-19 and perceived COVID-19 infectability supplement theory of planned behavior to explain iranians’ intention to get COVID-19 vaccinated. Vaccines.

[20] Du S (2023). The role of self-efficacy and self-care agency as mediating factors in the link between health literacy and health-promoting lifestyle among older adults post covid 19 era: a multiple mediator model. Geriatr Nurs.

[21] Aliakbari F, Tavassoli E, Mohammadalipour F. *The predictors of health literacy in patients with chronic obstructive pulmonary disease: an application of the social cognitive theory* Jour-nal of Clinical Nursing and Midwifery, 2020. 9(1): pp. 591–598.

[22] Tavousi M (2020). Development and validation of a short and easy-to-use instrument for measuring health literacy: the health literacy instrument for adults (HELIA). BMC Public Health.

[23] Budhathoki SS (2022). Use of the English health literacy questionnaire (HLQ) with health science university students in Nepal: a validity testing study. Int J Environ Res Public Health.

[24] Haghdoost AA et al. Iranian health literacy questionnaire (IHLQ): an instrument for measuring health literacy in Iran. Iran Red Crescent Med J, 2015. 17(6). 10.5812/ircmj.17(5)2015.25831 PMC4537788 26290752

[25] Tehrani Banihashemi S-A (2007). Health literacy and the influencing factors: a study in five provinces of Iran. Strides Dev Med Educ.

[26] Vasli P et al. The predictors of COVID-19 preventive health behaviors among adolescents: the role of health belief model and health literacy. J Public Health, 2022: p. 1–10. 10.1007/s10389-022-01808-x PMC9795447 36588661

[27] Kobryn M, Duplaga M (2024). Does health literacy protect against cyberchondria: a cross-sectional study?. Telemedicine e-Health.

[28] Shah VN (2018). Gender differences in diabetes self-care in adults with type 1 diabetes: findings from the T1D Exchange clinic registry. J Diabetes Complicat.

[29] Yousaf O, Grunfeld E, Hunter M. *review of the factors associated with delays in medical and psychological help-seeking among men. Health Psychology Review, 9 (2). pp. 264 – 76. ISSN 1743–7199 Link to official URL (if available)* 10.1080/17437199.2013.840954 26209212

[30] Seyedoshohadaee M (2016). The relationship between health literacy and self-care behaviors in patients with type 2 diabetes. Iran J Nurs Res.

[31] Khodabakhshi-Koolaee A (2016). The relationship of quality of life with health literacy in male patients with type II diabetes: a cross-sectional study in HARSIN city, 2015. J Diabetes Nurs.

[32] Aslan H, Mete B (2024). Health literacy level: Akyazı Example. Int J Health Manage Tourism.

[33] Beitler JJ (2010). Health literacy and health care in an inner-city, total laryngectomy population. Am J Otolaryngol.

[34] Saleh F et al. Diabetes education, knowledge improvement, attitudes and self-care activities among patients with type 2 diabetes in Bangladesh. Jundishapur J Health Sci, 2017. 9(1).

[35] Dashtian M (2017). Predicting factors affecting medication adherence and physical activity in patients with type-2 diabetes mellitus based on the theory of planned behavior. J School Public Health Inst Public Health Res.

[36] White KM (2012). An extended theory of planned behavior intervention for older adults with type 2 diabetes and cardiovascular disease. J Aging Phys Act.

[37] Rahmati-Najarkolaei F (2017). Determinants of lifestyle behavior in Iranian adults with prediabetes: applying the theory of planned behavior. Arch Iran Med.

[38] Zomahoun HTV et al. Predicting noninsulin antidiabetic drug adherence using a theoretical framework based on the theory of planned behavior in adults with type 2 diabetes: a prospective study. Medicine, 2016. 95(15). 10.1097/MD.0000000000002954 PMC4839786 27082543

[39] Grandieri A (2023). Relationship between people’s interest in medication adherence, health literacy, and self-care: an infodemiological analysis in the pre-and post-COVID-19 era. J Personalized Med.

[40] Lin JJ, Mann DM (2012). Application of persuasion and health behavior theories for behavior change counseling: design of the ADAPT (avoiding diabetes thru Action Plan Targeting) program. Patient Educ Couns.

[41] Paul B (2022). Theory of planned behaviour-based interventions in chronic diseases among low health-literacy population: protocol for a systematic review. Syst Reviews.

[42] Vasli P (2024). The predictors of COVID-19 preventive health behaviors among adolescents: the role of health belief model and health literacy. J Public Health.

[43] Duan Y (2022). Predicting hand washing, mask wearing and social distancing behaviors among older adults during the covid-19 pandemic: an integrated social cognition model. BMC Geriatr.

[44] Hayashi Y, Romanowich P, Hantula DA (2022). Predicting intention to take a COVID-19 vaccine in the United States: application and extension of theory of planned behavior. Am J Health Promotion.

[45] Okan O (2023). Health literacy as a social vaccine in the COVID-19 pandemic. Health Promot Int.

[46] Britanico JM. Association between COVID-19 Health Knowledge, Self-Efficacy and preventive behaviors among nursing students. Walden University; 2023.

[47] Didarloo A, et al. Assessment of factors affecting self-care behavior among women with type 2 diabetes in Khoy City Diabetes Clinic using the extended theory of reasoned action. Volume 9. Journal of School of Public Health & Institute of Public Health Research; 2011. 2.

[48] Bhaloo T, Juma M, Criscuolo-Higgins C (2018). A solution-focused approach to understanding patient motivation in diabetes self-management: gender differences and implications for primary care. Chronic Illn.

[49] Hrisos S (2009). Using psychological theory to understand the clinical management of type 2 diabetes in primary care: a comparison across two European countries. BMC Health Serv Res.

