# Supplementary material for: The mediating role of health literacy in the relationship between self-care and planned behavior against Covid-19
Source: BMC Infect Dis. 2024 Jun 20;24:608. doi: 10.1186/s12879-024-09513-8 (PMC11188506; doi:10.1186/s12879-024-09513-8)
Supplement: Supplementary file 1 — Supplementary Material 1 [file 12879_2024_9513_MOESM1_ESM.docx]

**The Health Literacy Instrument for Adults (HELIA)**

| N | Question | Likert scale | | | | |
| --- | --- | --- | --- | --- | --- | --- |
|  |  | 1 | 2 | 3 | 4 | 5 |
| 1 | Reading educational materials about health (booklets, pamphlets, and leaflets) is easy for me. |  |  |  |  |  |
| 2 | Reading written instructions from doctors, dentists and health workers about my illness is easy for me |  |  |  |  |  |
| 3 | Reading medical and dental forms (such as admissions, consent, filing, etc. in hospitals and medical  centers) is easy for me |  |  |  |  |  |
| 4 | Reading leaflets and instructions for laboratory testing, ultrasound or radiology is easy for me. |  |  |  |  |  |
| 5 | I can find health information from different sources when I need such information. |  |  |  |  |  |
| 6 | I can find health information about healthy eating. |  |  |  |  |  |
| 7 | I can find health information on mental health such as depression and stress. |  |  |  |  |  |
| 8 | I can find health information about a specific disease when I need to. |  |  |  |  |  |
| 9 | I can find health information for some health problems and diseases such as high blood pressure, high blood sugar and high lipid levels. |  |  |  |  |  |
| 10 | I can find health information about harmful effects of tobacco and smoking. |  |  |  |  |  |
| 11 | I can understand the recommendations for a healthy diet. |  |  |  |  |  |
| 12 | I can understand when my physician explains about my illness. |  |  |  |  |  |
| 13 | I can understand the meaning when reading medical forms (such as admissions, consents, filings, etc.) in hospitals and health centers. |  |  |  |  |  |
| 14 | I can understand signage guidelines in hospitals, clinics and health centers. |  |  |  |  |  |
| 15 | I can understand drug information on labels. |  |  |  |  |  |
| 16 | I can understand the risks, and benefits of drugs prescribed by my physician. |  |  |  |  |  |
| 17 | I can understand written information before testing, ultrasound or radiology. |  |  |  |  |  |
| 18 | I can evaluate health-related information on the Internet. |  |  |  |  |  |
| 19 | I can evaluate health-related information broadcast on television and radio. |  |  |  |  |  |
| 20 | I can assess the accuracy of health-related recommendations I receive from relatives and friends. |  |  |  |  |  |
| 21 | I can communicate trusted health information to others. |  |  |  |  |  |
| 22 | When facing an illness, I know where to go or with whom to speak. |  |  |  |  |  |
| 23 | When physician suggests that I should take antibiotic capsules three times a day I know that I should take one tablet every 8 h. |  |  |  |  |  |
| 24 | I do not cut my medications without my physician’s permission, even if symptoms disappear. |  |  |  |  |  |
| 25 | If anyone from my first-degree relatives develops cancer (such as prostate, breast, cervix, colon, etc.), I see a doctor to examine me. |  |  |  |  |  |
| 26 | I avoid doing or eating things that increase my blood pressure. |  |  |  |  |  |
| 27 | I visit my physician for regular checkups. |  |  |  |  |  |
| 28 | I am health-conscious in any situation. |  |  |  |  |  |
| 29 | If needed, I ask my physician or health care team questions about my disease. |  |  |  |  |  |
| 30 | I buy dairy products (milk, yoghurt, cheese, etc.) according to their fat percentage. |  |  |  |  |  |
| 31 | I avoid using substances that increase my weight. |  |  |  |  |  |
| 32 | I use a seat belt when driving. |  |  |  |  |  |
| 33 | I consider the food labels when shopping. |  |  |  |  |  |

**Questionnaire for theory of planned behavior in Covid-19 pandemic**

| N | Question | Likert scale | | | | |
| --- | --- | --- | --- | --- | --- | --- |
|  |  | 1 | 2 | 3 | 4 | 5 |
| 1 | If I follow preventive measures, I will be less vulnerable to the corona virus. |  |  |  |  |  |
| 2 | It is not difficult for me to follow preventive measures against the corona virus. |  |  |  |  |  |
| 3 | If I take preventive measures, I will be less anxious about the corona virus |  |  |  |  |  |
| 4 | People who are important to me think that I should act proactively |  |  |  |  |  |
| 5 | People who have an influence in my life think that I should take preventive actions |  |  |  |  |  |
| 6 | People whose opinion is important to me think that I should act proactively |  |  |  |  |  |
| 7 | I think preventive measures are easily implemented |  |  |  |  |  |
| 8 | I am sure that I can prevent myself from being infected with the corona virus |  |  |  |  |  |
| 9 | I am sure that I have enough information about the corona virus |  |  |  |  |  |
| 10 | Minimizing social activities and avoiding being in polluted and public areas |  |  |  |  |  |
| 11 | Use of a disposable medical mask when visiting public places or vehicles |  |  |  |  |  |
| 12 | Washing and keeping hands clean and having minimal contact with objects in public places |  |  |  |  |  |
| 13 | Avoiding touching the mouth, nose and eyes with unwashed hands and covering the mouth and nose with the elbow when sneezing or coughing. |  |  |  |  |  |
| 14 | Checking and controlling the health status according to the virus prevention protocols |  |  |  |  |  |
| 15 | Ensuring adequate ventilation at home and at work |  |  |  |  |  |
| 16 | Distance from others in public places to reduce the spread of infection |  |  |  |  |  |
| 17 | Vaccine injection in the recommended number of doses |  |  |  |  |  |

**Self-care questionnaire for Covid-19 pandemic**

| N | Question | Likert scale | | | | |
| --- | --- | --- | --- | --- | --- | --- |
|  |  | 1 | 2 | 3 | 4 | 5 |
| 1 | During the outbreak of the corona virus, I minimized my social activities (outside the home) and avoided being in contaminated and public areas. |  |  |  |  |  |
| 2 | During the outbreak of the corona virus, I used a disposable medical mask, especially in public places. |  |  |  |  |  |
| 3 | I have kept my hands clean and minimal contact with objects in public places during the coronavirus outbreak. |  |  |  |  |  |
| 4 | During the outbreak of the corona virus, I avoided touching my mouth, nose and eyes with unwashed hands and covered my mouth and nose with my elbow when sneezing or coughing. |  |  |  |  |  |
| 5 | During the outbreak of the corona virus, I checked and controlled my health according to the symptoms and medical guidelines announced. |  |  |  |  |  |
| 6 | During the outbreak of the Corona virus, I paid attention to the presence of adequate ventilation at home and at work. |  |  |  |  |  |
| 7 | During the outbreak of the corona virus, I kept a proper distance from others in public places. |  |  |  |  |  |
| 8 | I have injected the recommended number of doses of the vaccine during the corona virus outbreak. |  |  |  |  |  |
